# Rapid and sensitive detection of *Candidatus* Liberibacter asiaticus by loop mediated isothermal amplification combined with a lateral flow dipstick

**DOI:** 10.1186/1471-2180-14-86

**Published:** 2014-04-06

**Authors:** Luciano A Rigano, Florencia Malamud, Ingrid G Orce, Maria P Filippone, Maria R Marano, Alexandre Morais do Amaral, Atilio P Castagnaro, Adrian A Vojnov

**Affiliations:** 1 Instituto de Ciencia y Tecnología Dr. Cesar Milstein, Fundación Pablo Cassará, Consejo Nacional de Investigaciones Científicas y Técnicas (CONICET), Ciudad de Buenos Aires, Argentina; 2 Estación Experimental Agroindustrial Obispo Colombres (EEAOC)- CONICET, Instituto de Tecnología Agroindustrial del Noroeste Argentino (ITANOA), Las Talitas, Tucumán, Argentina; 3 Instituto de Biología Molecular y Celular de Rosario, Departamento de Microbiología, Facultad de Ciencias, Bioquímicas y Farmacéuticas, Universidad Nacional de Rosario, Rosario, Argentina; 4 Embrapa, Brasília, Distrito Federal, Brasil; 5 Department of Microbiology and Immunology, University of Otago, Dunedin, Otago, New Zealand; 6 Instituto de investigaciones fisiológicas y ecológicas vinculadas a la Agricultura (IFEVA-FAUBA), Universidad de Buenos Aires, Ciudad de Buenos Aires, Argentina

**Keywords:** Huanglongbing, *Candidatus* Liberibacter asiaticus, Diaphorina citri

## Abstract

**Background:**

Citrus Huanglongbing (HLB) is the most devastating bacterial citrus disease worldwide. Three *Candidatus* Liberibacter species are associated with different forms of the disease: *Candidatus* Liberibacter asiaticus, *Candidatus* Liberibacter americanus and *Candidatus* Liberibacter africanus. Amongst them, *Candidatus* Liberibacter asiaticus is the most widespread and economically important. These Gram-negative bacterial plant pathogens are phloem-limited and vectored by citrus psyllids. The current management strategy of HLB is based on early and accurate detection of *Candidatus* Liberibacter asiaticus in both citrus plants and vector insects. Nowadays, real time PCR is the method of choice for this task, mainly because of its sensitivity and reliability. However, this methodology has several drawbacks, namely high equipment costs, the need for highly trained personnel, the time required to conduct the whole process, and the difficulty in carrying out the detection reactions in field conditions.

**Results:**

A recent DNA amplification technique known as Loop Mediated Isothermal Amplification (LAMP) was adapted for the detection of *Candidatus* Liberibacter asiaticus. This methodology was combined with a Lateral Flow Dipstick (LFD) device for visual detection of the resulting amplicons, eliminating the need for gel electrophoresis. The assay was highly specific for the targeted bacterium. No cross-reaction was observed with DNA from any of the other phytopathogenic bacteria or fungi assayed. By serially diluting purified DNA from an infected plant, the sensitivity of the assay was found to be 10 picograms. This sensitivity level was proven to be similar to the values obtained running a real time PCR in parallel. This methodology was able to detect *Candidatus* Liberibacter asiaticus from different kinds of samples including infected citrus plants and psyllids.

**Conclusions:**

Our results indicate that the methodology here reported constitutes a step forward in the development of new tools for the management, control and eradication of this destructive citrus disease. This system constitutes a potentially field-capable approach for the detection of the most relevant HLB-associated bacteria in plant material and psyllid vectors.

## Background

Citrus Huanglongbing (HLB), literally from the Chinese “Yellow Shoot Disease”, is one of the most devastating diseases that threaten citrus production worldwide [1]. HLB is characterized by blotchy mottling with green areas on leaves. The infected shoots are stunted, and the branches gradually die as the disease progresses [2]. With the increase in disease severity, the yield is reduced and fruits quality is degraded. These affected fruit are smaller, lighter and highly acidic [2]. There are no curative procedures, and control of HLB consists of preventing trees from becoming infected and eradicating infected plants. Consequently, accurate and simple detection methods play a central role in reducing the incidence of HLB. The difficulty of correct diagnoses is partly because of the generic nature of HLB symptoms. The disease is sometimes misdiagnosed as nutrient deficiencies or other plant diseases [3].

Three fastidious α-Proteobacteria species of *Candidatus* Liberibacter, namely *Candidatus* Liberibacter asiaticus (*Las*), *Candidatus* Liberibacter americanus (*Lam*) and *Candidatus* Liberibacter africanus (*Laf*) are associated with HLB [1,2,4]. These three bacteria are associated with different forms of the disease and have worldwide distribution. *Las* has been reported to be the most widespread, destructive, and economically important, being present in Asia, Brazil and North America [1,2]. *Lam* and *Laf* are found in Brazil and Southern Africa respectively [1,3,5]. These pathogens are transmitted by grafting and by the sap-sucking psyllids *Diaphorina citri* in America and Asia, and *Trioza erytreae* in South Africa [6]. *Diaphorina citri* is considered the most serious pest of citrus worldwide, due primarily to its role as vector of *Las*[6]. The insect is present in America and Asia, and it spreads rapidly in residential and commercial plantings through natural ways, but also by commercial transport of infected plant material [6,7]. Worldwide, control of the psyllid *Diaphorina citri* as a vector is a central milestone in HLB management [6]. Therefore detection of infected insects is critical in preventing the spread of the disease [7].

Currently, the major initial detection procedure for *Las* is visual inspection based on disease symptoms in trees. Samples that are suspected to be positive are sent to diagnostic laboratories for secondary analysis. Several methodologies have been developed to detect *Las* in these samples, including serologic assays, electron microscopy, biological assays, DNA probes, Loop Mediated Isothermal Amplification, PCR and real-time PCR [1,8-16]. Many of these methods have the drawback of being time-consuming and requiring complex facilities. In addition to some of these approaches, detection of the pathogen in infected plants or vectors remain problematic [3]. In recent years, diagnosis of HLB by real time PCR methodologies has gained popularity due to its sensitivity and reliability [3,4,9,15], however real time PCR requires an expensive thermal cycler with a fluorescence detector, and highly trained personnel to perform assays and analyze data. These requirements reduce the suitability of real time PCR as an assay that can be performed “*in field*” (i.e., at the sampling site) or at border phytosanitary controls, places where complex facilities may not be available.

Loop-mediated isothermal amplification (LAMP) is a novel DNA amplification technique that amplifies DNA with high specificity, efficiency and rapidity under isothermal conditions [17]. LAMP is based on the principle of autocycling strand displacement DNA synthesis performed by the *Bst* DNA polymerase, for the detection of a specific DNA sequence [17]. The technique uses four to six primers that recognize six to eight regions of the target DNA and provides very high specificity [17,18]. Amplification can be carried out in a simple and inexpensive device like a water bath at temperatures between 60 to 65°C. LAMP produces large amounts of DNA [17] and shows high tolerance to biological contaminants [19], thereby simplifying sample preparation. Although LAMP products can be detected by gel electrophoresis, this procedure reduces the suitability for field applications.

As mentioned above, a LAMP methodology for the detection of *Las* has been previously reported [11]. That work focused on the detection of the DNA sequence of the *tuf*B-*sec*E-*nus*G-*rpl*KAJL-*rpo*B gene cluster present in the microorganism. The analysis of the amplification products was done by gel electrophoresis, or dot-blotting of the amplification products on a nylon membrane followed by staining with Mupid Blue, methods that are not compatible with field applications.

On our study, we target a hypothetical protein-coding sequence present in the genome of *Las* for the detection of this pathogen. To overcome the limitations associated with the gel electrophoresis, we coupled the LAMP amplification with a Lateral Flow Dipstick (LFD), which permits an accurate and straightforward detection of LAMP amplicons, eliminating the need of complex equipment and data analysis [20,21]. By using both LAMP and LFD technologies, this work describes the development of a new molecular diagnostic tool for the detection of *Las*.

## Results and discussion

In order to develop a successful HLB management strategy, methods for rapid detection of pathogens in the field are required. Such detection would allow early diagnosis of an infection focus before its spread. LAMP provides an ideal alternative for detection, as it requires a single incubation temperature and obviates the need for expensive thermal cyclers [17]. The combination of this isothermal DNA amplification technique with LFD devices has proven to be robust and successful in field-capable molecular diagnostics [20-22]. The recent sequencing of *Las* genome has uncovered new DNA sequences that can be used for pathogen detection through DNA amplification technologies [23]. Using an “*in silico*” approach, we found a hypothetical protein coding sequence, CLIBASIA_05175 [GenBank: ACT57606.1], which was predicted to be highly specific for *Las*. A BLASTn search [24] using the sequence of CLIBASIA_05175 as query, found homologs of this DNA sequence on *Candidatus* Liberibacter solanacearum, *Candidatus* Liberibacter americanus and *Liberibacter* crescens, however, no high level of sequence match was found between CLIBASIA_05175 and these sequences. An individual pairwise alignment between CLIBASIA_05175 and its BLASTn hits (Additional file 1: Figure S1) shows multiple mismatches on the primer binding regions, making unlikely a positive amplification with DNA from these other microorganisms. Accordingly, a DNA sample of *Candidatus* Liberibacter americanus did not produce positive amplification on the LAMP assay targeting CLIBASIA_05175 (Additional file 2: Figure S2).

**Figure 1:**
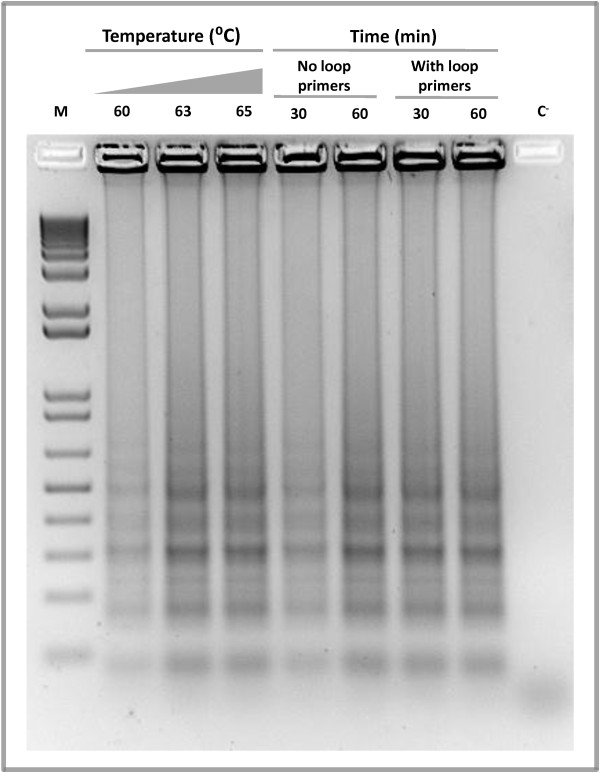
***Las*-LAMP reaction optimization.** Several temperature, time and primer combinations were applied to *Las*-LAMP to determine optimal reaction conditions. An aliquot of 10 μl of each *Las*-LAMP reaction was loaded into a 1.5% agarose gel. After electrophoresis, the gel was stained with ethidium bromide. C^-^: negative control without Template. M: 1 Kb plus DNA ladder (Invitrogen).

Reactions were optimized to establish the best assay conditions. To determine the optimal temperature, the reaction mixture was incubated at 60, 63 or 65°C for 60 minutes. With all tested temperatures, *Las*-LAMP products displayed the typical ladder-like pattern on gel electrophoresis with no amplification in the negative control lacking DNA (Figure 1). However, at 63 or 65°C the reaction was slightly more efficient than at 60°C, with no apparent difference between the first two. The specificity of the amplification was confirmed by sequencing (Additional file 3: Figure S3). As a result of this experiment, the temperature chosen for the assay was 65°C, as higher temperatures generally produce more stringent conditions for primer binding and greater amplification specificity [25]. We employed a thermal cycler, a water bath or an incubator to maintain the temperature necessary for the LAMP assay. The results indicated that all these devices were equally capable of producing efficient amplification (Additional file 4: Figure S4). Interestingly, a recent study shows that LAMP can be carried out using chemically driven heaters, a situation that could allow *Las*-LAMP amplifications in electricity-free locations [26].

Next we evaluated the effect of an improvement to the classic LAMP amplification, described previously [18]. Two additional primers named loop primers were added to the reaction mixture. The role of these oligonucleotides is to accelerate the reaction by providing more starting sites for the LAMP auto-cycling process. As shown in the Figure 1, the reaction containing loop primers and incubated at 65°C for 30 minutes performed as well as the reaction without loop primers and incubated for 60 minutes. Therefore, the optimal reaction conditions that were used in all subsequent experiments consisted of incubation at 65°C for 30 minutes with the inclusion of loop primers to the amplification mix.

**Figure 2:**
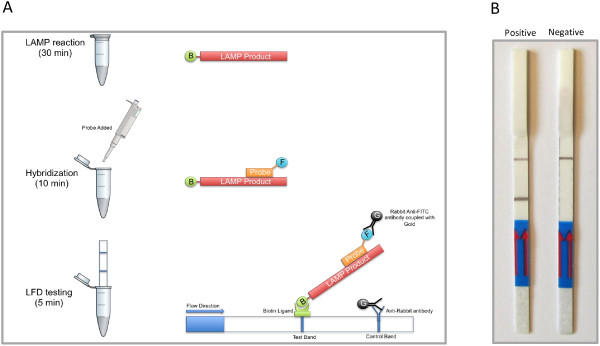
**Lateral flow dipstick *Las*-LAMP evaluation. A**. Lateral Flow Dipstick *Las*-LAMP procedure: LAMP reaction is performed using a biotinilated FIP primer. After 30 minutes of initial incubation at 65°C, a specific FITC-labelled probe is added to the reaction mixture and incubated for another 10 minutes at the same temperature. This step produces a dual labeled LAMP product. Finally, detection buffer containing Rabbit Anti-FITC antibodies coupled with colloidal gold is mixed with the reaction mixture, and the LFD strip is inserted into the tube. In a positive reaction, double labeled LAMP products migrates with the buffer flow and are retained at the Test Band by a biotin ligand. The gold coupled Anti-FICT antibody binds to the FITC molecule at the probe and a dark band develops over the time. In the case of a negative reaction no products are generated and such process does not have place. An Anti-Rabbit antibody at the Control Band retains some of the unbound gold-conjugated antibody and produces a Control Band that should be always visible. **B**. Evaluation of results using the Lateral Flow Dipstick device.

As previously mentioned, the analysis of LAMP amplicons by gel electrophoresis has several drawbacks, like the need of additional equipment, being a laborious and time consuming procedure, and requiring the use of the highly toxic ethidium bromide. These characteristics limit its use in field applications. To overcome these limitations, a generic lateral flow dipstick device (Milenia Biotec, Germany) was employed to detect the amplicons. This device detects biotin-labeled amplicons upon hybridization to a fluorescein isothiocyanate (FITC)-labeled DNA probe complexed with a gold-labeled anti-FITC antibody. The resulting triple complex moves by capillarity and is trapped by a biotin ligand at the test zone. As a result, the local gold concentration increases and a reddish-brown color line develops on the test zone during a positive reaction (Figure 2A).

When this methodology was used to detect *Las*-LAMP amplicons, we could distinguish two clear bands in the positive reaction. One of these bands was in the test zone and the other, which should be always present, was in the control zone. In contrast to the results with the positive reaction, in the negative control lacking DNA, only one band was visible and this was at the control zone (Figure 2B).

In order to determine the specificity of the *Las*-LAMP assay, purified DNA samples from several bacterial and fungal plant pathogens were evaluated. The results show that a positive reaction was obtained using DNA from plants infected with *Las*, but not with DNA from healthy plant material (Table 1, Additional file 5: Figure S5).

**Table 1:** **Specificity of the *Las*-LAMP assay**

**Species**	**Strain**	**Detection method**	
		**Gel**	**LFD**
*Candidatus* Liberibacter asiaticus	*	+	+
*Xylella fastidiosa*	9a5c	-	-
*Xanthomonas citri* subsp. *citri*	306	-	-
*Xanthomonas campestris* pv. *campestris*	8004	-	-
*Xanthomonas campestris* pv. *vesicatoria*	85-10	-	-
*Pseudomonas syringae*	DC3000	-	-
*Botrytis cinerea*	B-191	-	-
*Phytophthora citricola*	*	-	-
*Guignardia citricarpa*	*	-	-
*Elsinoe fawcettii*	*	-	-
Healthy Orange	N/A	-	-
Healthy *Citrus limon*	N/A	-	-
Healthy *Diaphorina citri*	N/A	-	-

For each sample *Las*-LAMP reaction was performed in triplicate. Gel: gel electrophoresis. LFD: lateral flow dipstick. +: Positive reaction. -: Negative reaction. *Performed with DNA from an infected plant without symptoms of other disease. N/A: Not applicable.

Negative results were obtained with DNA from other common citrus and plant pathogens, indicating a high level of specificity (Table 1). This specificity is likely due to the DNA region selected for amplification and also the nature of LAMP, which recognizes eight regions in the target DNA. LFD detection of the resulting amplicons adds another layer of specificity, because in order to be detected, the amplicons must hybridize specifically with the probe. Since genomic data is not available, and we have not analyzed samples of the related pathogen *Candidatus* Liberibacter africanus in this work, we can not exclude the possibility of a positive reaction with DNA from this pathogen.

The *Las*-LAMP assay sensitivity was determined using serial dilutions of total purified DNA from a *Las* positive plant. The same samples were evaluated in parallel by previously described real time PCR procedure [3] in order to compare sensitivities of both methods. Both gel electrophoresis and LFD detection of *Las*-LAMP amplicons showed the same detection limit of 10 picograms of DNA (Table 2, Additional file 5: Figure S5). Interestingly, this detection limit was similar to that of the real time PCR assay. These results demonstrate that the fast and straightforward detection alternative that we describe here is at least as sensitive as the more complex and expensive approach of real time PCR.

**Table 2:** **Comparison between *Las*-LAMP and real time PCR assay sensitivity from DNA purified from a *Candidatus *** **Liberibacter asiaticus positive plant**

**Detection method**	**Purified DNA from a *Las *** **positive citrus plant**						
	**100 ng**	**10 ng**	**1 ng**	**100 pg**	**10 pg**	**1 pg**	**100 fg**
*Las*-LAMP Gel	+	+	+	+	+	-	-
*Las*-LAMP FLD	+	+	+	+	+	-	-
Real time PCR	+	+	+	+	+	-	-

For each dilution the *Las*-LAMP reaction was performed in triplicate. Gel: gel electrophoresis. LFD: lateral flow dipstick. +: Positive reaction. -: Negative reaction. Real time PCR have been scored as positive if amplification could be detected during the reaction time.

**Figure 3:**
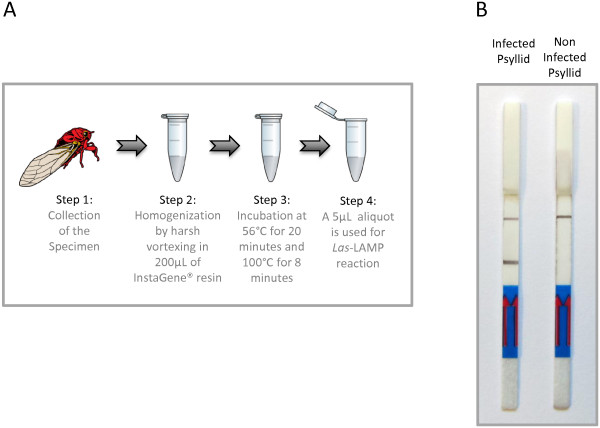
**Fast detection of *Las *from *Diaphorina citri. *A**. Fast psyllid sample preparation procedure: The specimen is grinded by harsh vortexing in 200 μL of the PCR inhibitor chelator matrix InstaGene® (BIORAD™). After that, the sample is incubated at 56°C for 20 minutes to activate the matrix binding groups and subjected to a final incubation at 100°C for 8 minutes to disrupt cell material. A 5-μL aliquot is then used for *Las*-LAMP. **B**. *Las*-LAMP results from an infected psyllid (left) and an uninfected psyllid (right). On Figure 3A, the insect image is representative of a general flying insect for illustration purposes and it is not intended to represent an actual *Diaphorina citri* psyllid.

The ability of this technique to detect *Las* in the vector psyllid, *Diaphorina citri* was evaluated using a simple and fast sample preparation method (Figure 3A). Briefly, one *Las*-infected insect was homogenized by vortexing in presence of InstaGene resin (BIORAD®), incubated at 56°C for 20 minutes to activate the resin chelating groups and then incubated for 8 minutes at 100°C in order to destroy cellular structures and release the nucleic acids. When 5 μL of this lysate was added to the *Las*-LAMP reaction mixture a positive amplification was detected by gel electrophoresis (data not shown) and LFD (Figure 3B). No amplification was detected with a non-infected insect. The same fast sample preparation method was evaluated for the detection of *Las* in leaf samples, but it did not perform well (data not shown). We are not certain of the reasons for this difference, but it might be related to the low number of bacteria present in the leaves compared with the psyllids and the presence of more potential amplification inhibitors present in leaf material.

Positive amplification was achieved with several field samples of *Las* extracted for DNA, including infected trees and psyllids (Table 3) demonstrating the robustness of the assay. However, further studies with a more diverse set of field samples, including those from worldwide locations, should be performed in the future, to confirm that the methodology efficiently detects *Las* from different geographical origins.

**Table 3:** **Samples of *Candidatus *** **Liberibacter asiaticus positive material used to evaluate the *Las*-LAMP assay**

**Species**	**Sample name**	**Origin**		**Detection method**	
		**Host**	**Country**	***Las*-LAMP gel**	***Las*-LAMP LFD**
*Candidatus* Liberibacter asiaticus	CLAS-12	Orange	Brazil	+	+
*Candidatus* Liberibacter asiaticus	CLAS-13	Orange	Brazil	+	+
*Candidatus* Liberibacter asiaticus	CLAS-14	Orange	Brazil	+	+
*Candidatus* Liberibacter asiaticus	CLAS-15	Orange	Brazil	+	+
*Candidatus* Liberibacter asiaticus	CLAS-16	Orange	Brazil	+	+
*Candidatus* Liberibacter asiaticus	CLAS-17	Orange	Brazil	+	+
*Candidatus* Liberibacter asiaticus	CLAS-18	Orange	Brazil	+	+
*Candidatus* Liberibacter asiaticus	CLAS-19	Orange	Brazil	+	+
*Candidatus* Liberibacter asiaticus	CLAS-20	Orange	Brazil	+	+
*Candidatus* Liberibacter asiaticus	CLAS-21	Orange	Brazil	+	+
*Candidatus* Liberibacter asiaticus	CHICHALAS-1	Psyllid	Brazil	+	+
*Candidatus* Liberibacter asiaticus	CHICHALAS-2	Psyllid	Brazil	+	+
*Candidatus* Liberibacter asiaticus	CHICHALAS-3	Psyllid	Brazil	+	+
*Candidatus* Liberibacter asiaticus	CHICHALAS-4	Psyllid	Brazil	+	+
*Candidatus* Liberibacter asiaticus	CHICHALAS-5	Psyllid	Brazil	+	+
*Candidatus* Liberibacter asiaticus	CHICHALAS-6	Psyllid	Brazil	+	+
*Candidatus* Liberibacter asiaticus	CHICHALAS-7	Psyllid	Brazil	+	+
*Candidatus* Liberibacter asiaticus	CHICHALAS-8	Psyllid	Brazil	+	+
*Candidatus* Liberibacter asiaticus	CHICHALAS-9	Psyllid	Brazil	+	+
*Candidatus* Liberibacter asiaticus	CHICHALAS-10	Psyllid	Brazil	+	+
*Candidatus* Liberibacter asiaticus	CHICHALAS-11	Psyllid	Brazil	+	+

For each isolate the *Las*-LAMP reaction was performed in triplicate. Gel: gel electrophoresis. LFD: lateral flow dipstick. +: Positive reaction. -: Negative reaction.

The combination of LAMP with a LFD amplicon detection system, allows for detection of *Las* at a speed not previously reported, taking just 45 minutes from the start of the amplification to the evaluation of the results. This characteristic combined with the capability to be carried out in a low resource setting makes the method presented here a powerful diagnostic tool for HLB.

## Conclusions

In this work, we targeted a sequence on the gene CLIBASIA_05175 to develop and validate a LAMP methodology for detection of *Las* in both host plants and vector insects. To the best of our knowledge, this study constitutes the first report of an isothermal-lateral flow dipstick coupled detection system for diagnosis of HLB with the potential for “*in field*” applications. This alternative approach was demonstrated to be fast, sensitive and specific in different kinds of samples including leaf material or psyllids. The results of this study provide evidence that this LAMP-based method can be reliably integrated into the HLB management as a tool for faster diagnostics.

## Methods

### Biological samples

Citrus leaf samples were collected from *Las* symptomatic and asymptomatic sweet orange (*Citrus sinensis*) trees in orchards from Sao Paulo state, Brazil, during summer and transported at room temperature in a sealed container. The samples were maintained a 4°C until they were used for DNA purification, typically 1–2 days after collection. Psyllids were collected and stored submerged in 75% ethanol until DNA extraction, typically 1–2 days after collection.

### DNA extraction

Midribs were separated from leaf samples and cut into smaller pieces. DNA was extracted using the Wizard® Genomic DNA purification Kit, Promega, Madison, WI, USA, according the manufacturer’s instructions and resuspended in 100 μL of ultrapure water. The presence of *Las* in the samples was confirmed by real time PCR as described previously [3]. DNA samples from *Diaphorina citri* were prepared as follows, a single infected insect was homogenized by vortexing in presence of 200 μL of InstaGene™ resin (BIORAD®), incubated at 56°C for 20 minutes to activate the resin chelating groups and then incubated for 8 minutes at 100°C in order to destroy cellular structures and release the nucleic acids. Five microliters of this preparation were added to the *Las*-LAMP reaction mix as template.

### Computational analysis

In order to find a suitable DNA region on the genome of *Candidatus* Liberibacter asiaticus allowing a specific detection of the microorganism, we manually selected hypothetical protein coding regions from the genome for BLASTn searches [24]. The rationale for this is that hypothetical proteins are open reading frames with low or no homology to known protein coding genes. Sequences showing lower homology with sequences from other organisms were selected.

### LAMP reaction

**Figure 4:**
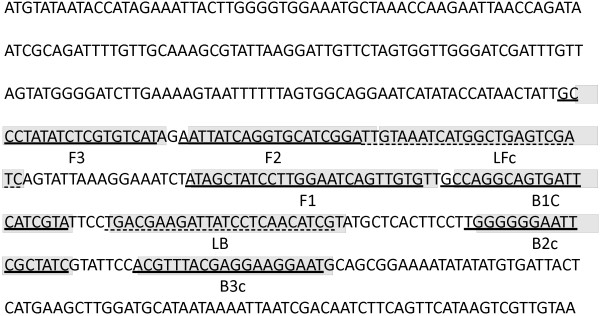
**Localization of target sequences used for primer construction.** Target sequences used for LAMP primer design are underlined and shaded over the whole sequence of the gene CLIBASIA_05175. Solid lines correspond to F3, F2, F1 B1c, B2c and B3c regions. Dashed line corresponds to loop primers binding regions LFc and LB.

Oligonucleotide LAMP primers were designed according to the published sequence of the gene CLIBASIA_05175 [GenBank: ACT57606.1], from the *Candidatus* Liberibacter asiaticus genome. The software Primer Explorer version 4 (Net Laboratory, Tokyo, Japan) was used to target the middle region of the gene (Figure 4), resulting in primers *Las*-F3, *Las*-B3, *Las*-FIP and *Las*-BIP (Table 4). In addition, a set of two Loop primers, *Las*-LF and *Las*-LB was generated for reaction acceleration (Table 4). The *Las*-LAMP assay was performed using a dry thermal block with a 0.5-mL PCR tube holder. The final LAMP conditions used were as follows, 40 pmol each of primers *Las*-FIP and *Las*-BIP, 5 pmol each of outer primers *Las*-F3 and *Las*-B3, 20 pmol each of loop primers *Las*-LF and *Las*-LB, 8 U of *Bst* DNA polymerase, 4.5 mM MgSO_4_, 1.4 mM of dNTP mix, 20 mM Tris–HCl (pH 8.8), 10 mM KCl, 10 mM (NH_4_)_2_SO_4_, 0.1% Triton X-100 and 1.6 M betaine, in a final volume of 25 μL including the template. This reaction mix was incubated at 65°C for 30 minutes.

**Table 4:** **Sequences of primers used for the *Las*-LAMP assay**

**Primer name**	**Type**	**Sequence (5′-3′)**	**Length**
*Las*-F3	F3	GCCCTATATCTCGTGTCAT	19 mer
*Las*-B3	B3	ATTCCTTCCTCGTAAACGT	19 mer
*Las*-FIP	FIP (F1c + F2)	CACAACTGATTCCAAGGATAGCT-	44 mer
		ATAATTATCAGGTGCATCGGA	
*Las*-BIP	BIP (B1c + B2)	GCCAGGCAGTGATTCATCGTAG-	39 mer
		ATAGCGAATTCCCCCCA	
*Las*-LF	LF	GATCGACTCAGCCATGATTTACAA	24 mer
*Las*-LB	LB	TGACGAAGATTATCCTCAACATCG	24 mer

### Analysis of LAMP products

The products of amplification were subjected to electrophoresis at 85 V for 50 minutes on a 1.5% agarose gel, followed by ethidium bromide staining. To confirm the specificity of the product some bands were cut and sequenced. The sequences obtained were used as queries to perform BLAST searches [24] in order to confirm identity.

Lateral flow dipstick analyses of *Las*-LAMP products were performed as described previously [20,21]. Briefly, a biotin-labeled FIP primer was used in the *Las*-LAMP reaction. All other components in the reaction mix remained the same as described above, resulting in biotin-labeled *Las*-LAMP amplicons. A 5′ FITC-labeled DNA probe (5′-FITC-CTCAACATCGTATGCTCACTT-3′) was designed to hybridize in the region between the *Las*-FIP and *Las*-BIP primers. Twenty picomol of this probe were added at the end of the *Las*-LAMP amplification reaction and incubated at 65°C for 10 minutes to allow for hybridization. After the hybridization step, 10 μL of this product was mixed with 150 μL of the strip assay buffer (Milenia® HybriDetect, MGHD1, Milenia Biotec, Germany). Finally the LFD strip was submerged into the mixture, and the results were visualized after 5 minutes.

### Sensitivity of LAMP and real time PCR

In order to estimate the sensitivity of the *Las*-LAMP assay, purified DNA from a *Las* infected plant was serially diluted and 1 μL aliquots of these dilutions were used as template for *Las*-LAMP and real time PCR. *Las*-LAMP reactions were performed as mentioned above, and real time PCR was carried out as described previously [3], in a Step One™ real time PCR system (Applied Biosystems®).

## Competing interests

The authors declare that they have no competing interests.

## Authors’ contributions

LAR designed the experiments, performed the experimental work and wrote the manuscript; FM performed experimental work and wrote the manuscript, IGO and MPF performed experiments with DNA from *Candidatus* Liberibacter americanus. MRM, AMDA and APC contributed to coordinate the study and wrote the manuscript; AAV participated in the analysis and interpretation of the data and wrote the manuscript. All authors read and approved the final manuscript.

## Supplementary Material

Additional file 1: Figure S1 Pairwise alignment between CLIBASIA_05175 and related sequences from a BLASTn search. A. BLASTn pairwise alignment between CLIBASIA_05175 (green) and a related sequence from *Candidatus* Liberibacter solanacearum (black). *Las*-LAMP primer binding sites are highlighted in yellow and cyan. B. BLASTn pairwise alignment between CLIBASIA_05175 (green) and a related sequence from *Candidatus* Liberibacter americanus (black). *Las*-LAMP primer binding sites are highlited in yellow and cyan. C. BLASTn pairwise alignment between CLIBASIA_05175 (green) and a related sequence from *Candidatus* crescens (black). *Las*-LAMP primer binding sites are highlighted in yellow and in cyan. Click here for file

Additional file 2: Figure S2 Evaluation of *Candidatus* Liberibacter americanus DNA by *Las*-LAMP. Purified DNA from plants infected with *Candidatus* Liberibacter asiaticus (*Las*) or *Candidatus* Liberibacter americanus (*Lam*) were used as templates for the *Las*-LAMP amplification reaction. A. Amplification products analyzed by gel electrophoresis. B. Amplification products analyzed using a lateral flow dipstick. C^-^: negative control without template. M: 1 Kb plus DNA ladder (Invitrogen®), the size of the bands is, from bottom to top: 100 bp, 200 bp, 300 bp, 400 bp, 500 bp, 650 bp, 850 bp, 1000 bp, 1650 bp, 2000 bp and increments of 1000 bp up to 12000 bp. Click here for file

Additional file 3: Figure S3 Pairwise alignment between CLIBASIA_05175 and the sequence of a *Las*-LAMP amplification product. A *Las*-LAMP amplification product band was Analyzed by sequencing. The sequence corresponding to the amplification product (red), from F2 to B2c, has been subjected to a pairwise alignment against CLIBASIA_05175 (green). Click here for file

Additional file 4: Figure S4 Evaluation of different heating devices on *Las*-LAMP amplification. A thermal cycler (1), a water bath (2) or an incubator (3), were used to maintain the temperature required for *Las*-LAMP amplification reaction. A. Amplification products were Analyzed by gel electrophoresis. B. Amplification products Analyzed using a lateral flow dipstick. C^-^: negative control without Template. M: 1 Kb plus DNA ladder (Invitrogen®), the size of the bands is from bottom to top: 100 bp, 200 bp, 300 bp, 400 bp, 500 bp, 650 bp, 850 bp, 1000 bp, 1650 bp, 2000 bp and increments of 1000 bp up to 12000 bp. Click here for file

Additional file 5: Figure S5 Images of gel electrophoresis and lateral flow dipsticks corresponding to Table 1 and Table 2. A. Images of gel electrophoresis (left) and lateral flow dipsticks (right) corresponding to samples in Table 1. 1. *Candidatus* Liberibacter asiaticus, 2. *Xylella fastidiosa*, 3. *Xanthomonas campestris* pv. *campestris*, 4. *Xanthomonas campestris* pv. *vesicatoria*, 5. *Pseudomonas syringae*, 6. *Botrytis cinerea*, 7. *Phytophthora citricola*, 8. *Guignardia citricarpa*, 9. *Elsinoe fawcettii*, 10. Healthy Orange, 11. Healty *Citrus limon*, 12. Healty *Diaphorina citri*. B. Images of gel electrophoresis (left) and lateral flow dipsticks (right) corresponding to samples in Table 2. 1. 100 ng DNA, 2. 10 ng DNA, 3. 1 ng DNA, 4. 100 pg DNA, 5. 10 pg DNA, 6. 1 pg DNA, 7. 100 fg DNA. For all gels, M: 1 Kb plus DNA ladder (Invitrogen®), the size of the bands is from bottom to top: 100 bp, 200 bp, 300 bp, 400 bp, 500 bp, 650 bp, 850 bp, 1000 bp, 1650 bp, 2000 bp and increments of 1000 bp up to 12000 bp. Click here for file

## References

[B1] Gottwald TR Current epidemiological understanding of citrus Huanglongbing Annu Rev Phytopathol 2010 48 119 139 10.1146/annurev-phyto-073009-114418 20415578

[B2] Wang N Trivedi P Citrus huanglongbing: a newly relevant disease presents unprecedented challenges Phytopathology 2013 103 7 652 665 10.1094/PHYTO-12-12-0331-RVW 23441969

[B3] Li W Hartung JS Levy L Quantitative real-time PCR for detection and identification of Candidatus Liberibacter species associated with citrus huanglongbing J Microbiol Methods 2006 66 1 104 115 10.1016/j.mimet.2005.10.018 16414133

[B4] Morgan JK Zhou L Li W Shatters RG Keremane M Duan YP Improved real-time PCR detection of ‘Candidatus Liberibacter asiaticus’ from citrus and psyllid hosts by targeting the intragenic tandem-repeats of its prophage genes Mol Cell Probes 2012 26 2 90 98 10.1016/j.mcp.2011.12.001 22245034

[B5] Do Carmo Teixeira D Luc Danet J Eveillard S Cristina Martins E De Jesus Junior WC Takao Yamamoto P Aparecido Lopes S Beozzo Bassanezi R Juliano Ayres A Saillard C Bove JM Citrus huanglongbing in Sao Paulo State, Brazil: PCR detection of the ‘Candidatus’ Liberibacter species associated with the disease Mol Cell Probes 2005 19 3 173 179 10.1016/j.mcp.2004.11.002 15797817

[B6] Grafton-Cardwell EE Stelinski LL Stansly PA Biology and management of Asian citrus psyllid, vector of the huanglongbing pathogens Annu Rev Entomol 2013 58 413 432 10.1146/annurev-ento-120811-153542 23317046

[B7] Manjunath KL Halbert SE Ramadugu C Webb S Lee RF Detection of ‘Candidatus Liberibacter asiaticus’ in Diaphorina citri and its importance in the management of citrus huanglongbing in Florida Phytopathology 2008 98 4 387 396 10.1094/PHYTO-98-4-0387 18944186

[B8] Garnier M Martin-Gros G Bove JM Monoclonal antibodies against the bacterial-like organism associated with citrus greening disease Ann Inst Pasteur Microbiol 1987 138 6 639 650 10.1016/0769-2609(87)90142-6 3331293

[B9] JM B Huanglongbing: a destructive, newly emerging, century-old disease of citrus J Plant Pathol 2006 88 7 37

[B10] Hocquellet A Bove JM Garnier M Production and evaluation of non-radioactive probes for the detection of the two ‘Candidatus Liberobacter’ species associated with citrus huanglongbing (greening) Mol Cell Probes 1997 11 6 433 438 10.1006/mcpr.1997.0140 9500815

[B11] Okuda MMM Tanaka Y Characterization of the tufB-secE-nusG-rplKAJL-rpoB Gene Cluster of the Citrus Greening Organism and Detection by Loop-Mediated Isothermal Amplification Plant Dis 2005 89 7 705 711 10.1094/PD-89-0705 30791239

[B12] Teixeira DC Saillard C Couture C Martins EC Wulff NA Eveillard-Jagoueix S Yamamoto PT Ayres AJ Bove JM Distribution and quantification of Candidatus Liberibacter americanus, agent of huanglongbing disease of citrus in Sao Paulo State, Brasil, in leaves of an affected sweet orange tree as determined by PCR Mol Cell Probes 2008 22 3 139 150 10.1016/j.mcp.2007.12.006 18400468

[B13] Jagoueix S Bove JM Garnier M PCR detection of the two ‘Candidatus’ Liberobacter species associated with greening disease of citrus Mol Cell Probes 1996 10 1 43 50 10.1006/mcpr.1996.0006 8684375

[B14] Fujikawa T Iwanami T Sensitive and robust detection of citrus greening (huanglongbing) bacterium “Candidatus Liberibacter asiaticus” by DNA amplification with new 16S rDNA-specific primers Mol Cell Probes 2012 26 5 194 197 10.1016/j.mcp.2012.06.001 22728344

[B15] Lin H Chen C Doddapaneni H Duan Y Civerolo EL Bai X Zhao X A new diagnostic system for ultra-sensitive and specific detection and quantification of Candidatus Liberibacter asiaticus, the bacterium associated with citrus Huanglongbing J Microbiol Methods 2010 81 1 17 25 10.1016/j.mimet.2010.01.014 20096734

[B16] Kim JS Wang N Characterization of copy numbers of 16S rDNA and 16S rRNA of Candidatus Liberibacter asiaticus and the implication in detection in planta using quantitative PCR BMC Res Notes 2009 2 37 10.1186/1756-0500-2-37 19284534 PMC2663771

[B17] Notomi T Okayama H Masubuchi H Yonekawa T Watanabe K Amino N Hase T Loop-mediated isothermal amplification of DNA Nucleic Acids Res 2000 28 12 E63 10.1093/nar/28.12.e63 10871386 PMC102748

[B18] Nagamine K Hase T Notomi T Accelerated reaction by loop-mediated isothermal amplification using loop primers Mol Cell Probes 2002 16 3 223 229 10.1006/mcpr.2002.0415 12144774

[B19] Kaneko H Kawana T Fukushima E Suzutani T Tolerance of loop-mediated isothermal amplification to a culture medium and biological substances J Biochem Biophys Methods 2007 70 3 499 501 10.1016/j.jbbm.2006.08.008 17011631

[B20] Kiatpathomchai W Jaroenram W Arunrut N Jitrapakdee S Flegel TW Shrimp Taura syndrome virus detection by reverse transcription loop-mediated isothermal amplification combined with a lateral flow dipstick J Virol Methods 2008 153 2 214 217 10.1016/j.jviromet.2008.06.025 18662723

[B21] Khunthong S Jaroenram W Arunrut N Suebsing R Mungsantisuk I Kiatpathomchai W Rapid and sensitive detection of shrimp yellow head virus by loop-mediated isothermal amplification combined with a lateral flow dipstick J Virol Methods 2013 188 1–2 51 56 23219929 10.1016/j.jviromet.2012.11.041

[B22] Rigano LA Marano MR Castagnaro AP Do Amaral AM Vojnov AA Rapid and sensitive detection of Citrus Bacterial Canker by loop-mediated isothermal amplification combined with simple visual evaluation methods BMC Microbiol 2010 10 176 10.1186/1471-2180-10-176 20565886 PMC2895605

[B23] Duan Y Zhou L Hall DG Li W Doddapaneni H Lin H Liu L Vahling CM Gabriel DW Williams KP Dickerman A Sun Y Gottwald T Complete genome sequence of citrus huanglongbing bacterium, ‘Candidatus Liberibacter asiaticus’ obtained through metagenomics Mol Plant Microbe Interact 2009 22 8 1011 1020 10.1094/MPMI-22-8-1011 19589076

[B24] Altschul SF Gish W Miller W Myers EW Lipman DJ Basic local alignment search tool J Mol Biol 1990 215 3 403 410 2231712 10.1016/S0022-2836(05)80360-2

[B25] Tindall KR Kunkel TA Fidelity of DNA synthesis by the Thermus aquaticus DNA polymerase Biochemistry 1988 27 16 6008 6013 10.1021/bi00416a027 2847780

[B26] LaBarre P Hawkins KR Gerlach J Wilmoth J Beddoe A Singleton J Boyle D Weigl B A simple, inexpensive device for nucleic acid amplification without electricity-toward instrument-free molecular diagnostics in low-resource settings PLoS One 2011 6 5 e19738 10.1371/journal.pone.0019738 21573065 PMC3090398

