# Supplementary figures and images for: Rapid and sensitive detection of Candidatus Liberibacter asiaticus by loop mediated isothermal amplification combined with a lateral flow dipstick
Source: BMC Microbiol. 2014 Apr 6;14:86. doi: 10.1186/1471-2180-14-86 (PMC4021466; doi:10.1186/1471-2180-14-86)

## Slide 1
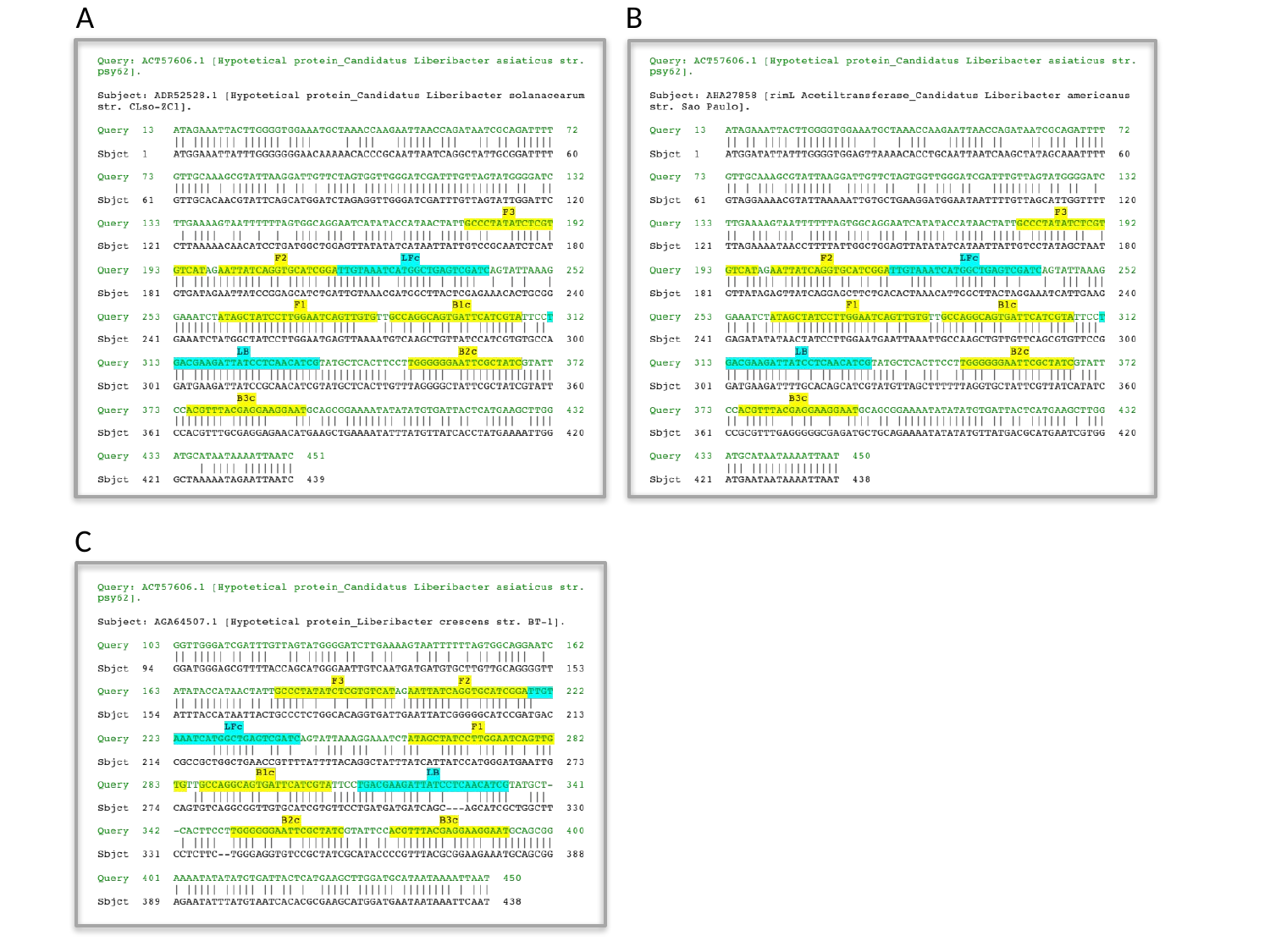

B
A
C

Supplement: Additional file 1: Figure S1 — Pairwise alignment between CLIBASIA_05175 and related sequences from a BLASTn search. A. BLASTn pairwise alignment between CLIBASIA_05175 (green) and a related sequence from Candidatus Liberibacter solanacearum (black). Las-LAMP primer binding sites are highlighted in yellow and cyan. B. BLASTn pairwise alignment between CLIBASIA_05175 (green) and a related sequence from Candidatus Liberibacter americanus (black). Las-LAMP primer binding sites are highlited in yellow and cyan. C. BLASTn pairwise alignment between CLIBASIA_05175 (green) and a related sequence from Candidatus crescens (black). Las-LAMP primer binding sites are highlighted in yellow and in cyan. [file 1471-2180-14-86-S1.pptx]

## Slide 1
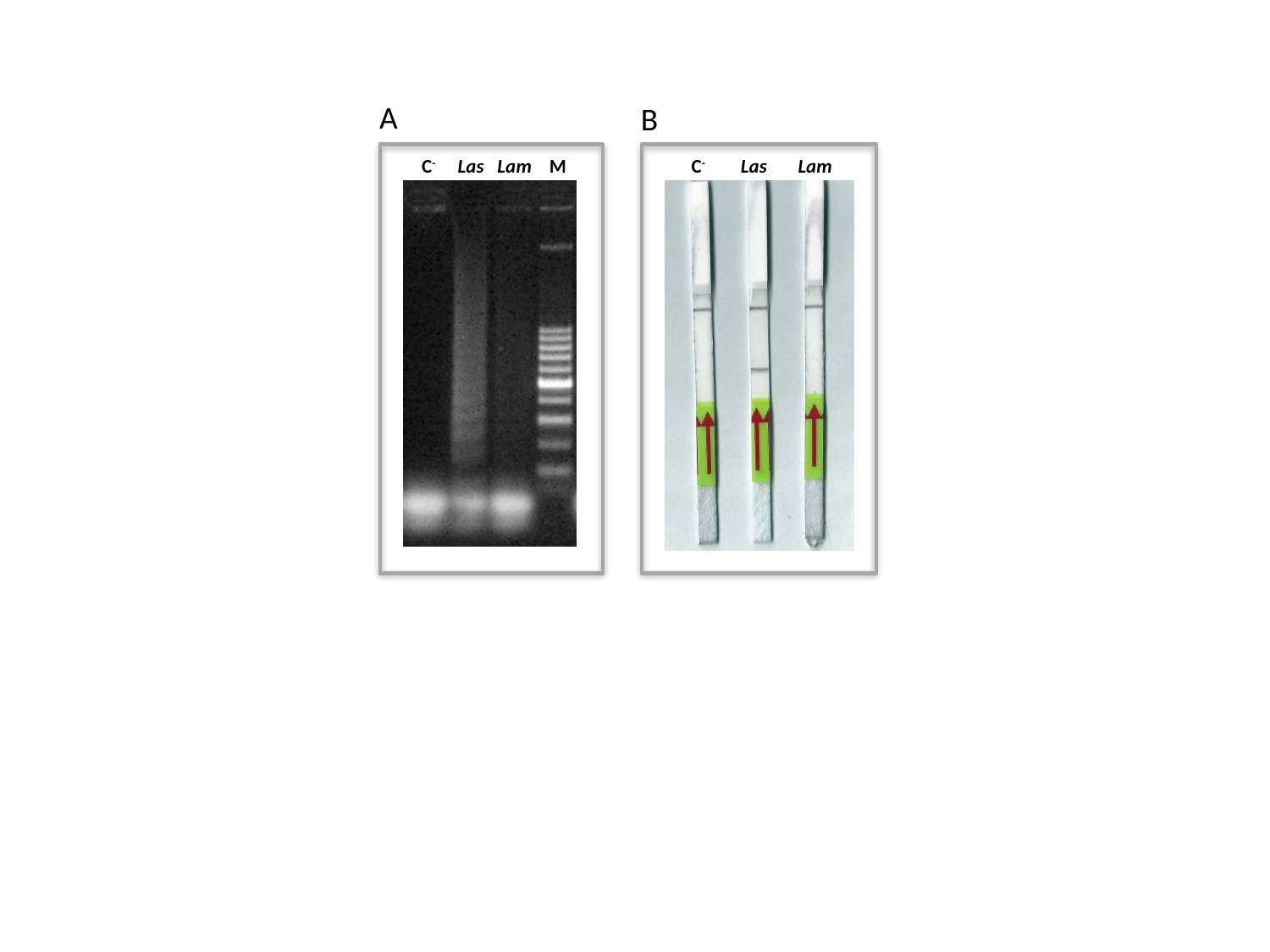

A
B
 C- Las Lam
 C- Las Lam M

Supplement: Additional file 2: Figure S2 — Evaluation of Candidatus Liberibacter americanus DNA by Las-LAMP. Purified DNA from plants infected with Candidatus Liberibacter asiaticus (Las) or Candidatus Liberibacter americanus (Lam) were used as templates for the Las-LAMP amplification reaction. A. Amplification products analyzed by gel electrophoresis. B. Amplification products analyzed using a lateral flow dipstick. C-: negative control without template. M: 1 Kb plus DNA ladder (Invitrogen®), the size of the bands is, from bottom to top: 100 bp, 200 bp, 300 bp, 400 bp, 500 bp, 650 bp, 850 bp, 1000 bp, 1650 bp, 2000 bp and increments of 1000 bp up to 12000 bp. [file 1471-2180-14-86-S2.pptx]

## Slide 1
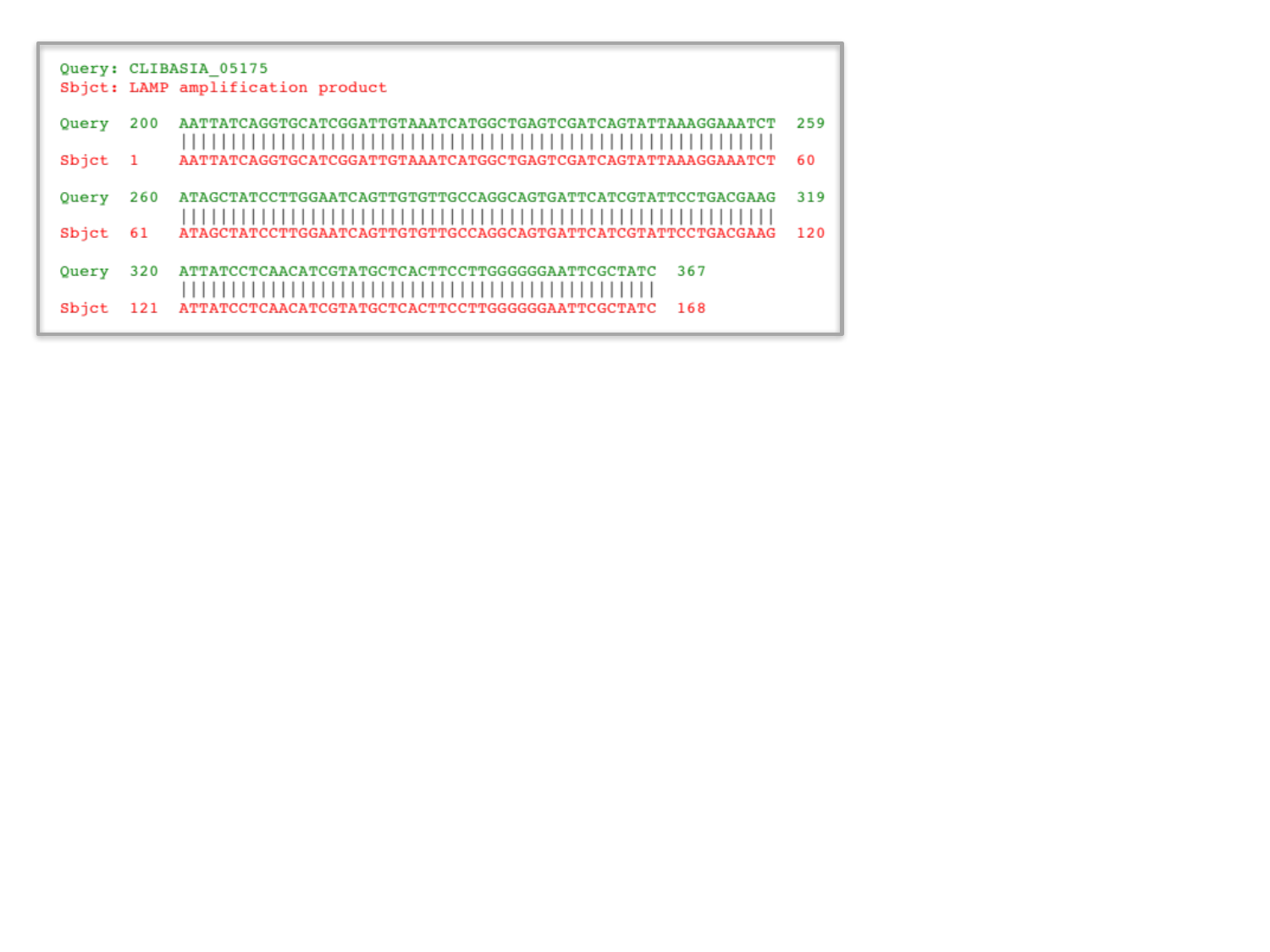

Supplement: Additional file 3: Figure S3 — Pairwise alignment between CLIBASIA_05175 and the sequence of a Las-LAMP amplification product. A Las-LAMP amplification product band was Analyzed by sequencing. The sequence corresponding to the amplification product (red), from F2 to B2c, has been subjected to a pairwise alignment against CLIBASIA_05175 (green). [file 1471-2180-14-86-S3.pptx]

## Slide 1
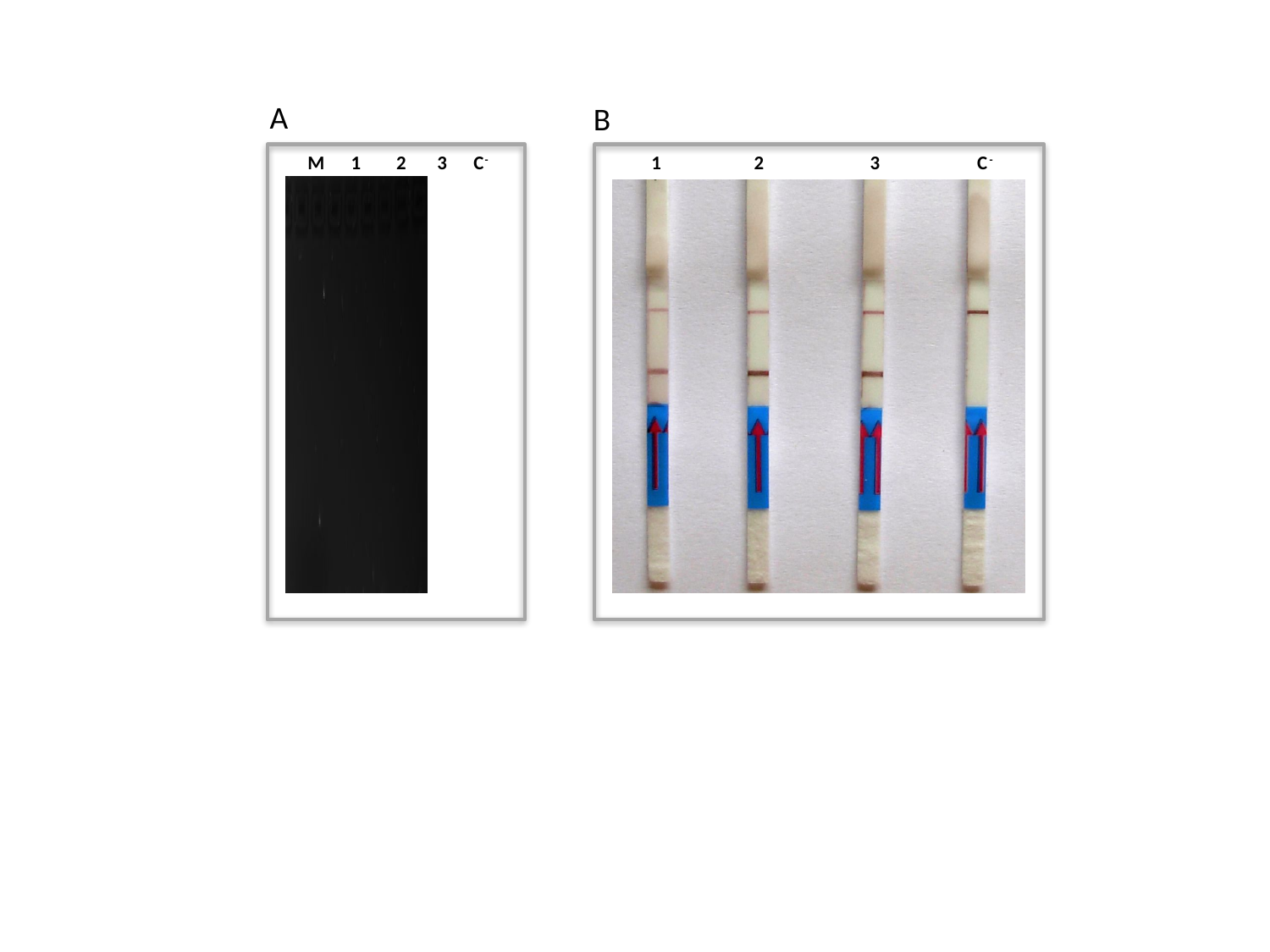

A
B
M 1 2 3 C-
 1 2 3 C-

Supplement: Additional file 4: Figure S4 — Evaluation of different heating devices on Las-LAMP amplification. A thermal cycler (1), a water bath (2) or an incubator (3), were used to maintain the temperature required for Las-LAMP amplification reaction. A. Amplification products were Analyzed by gel electrophoresis. B. Amplification products Analyzed using a lateral flow dipstick. C-: negative control without Template. M: 1 Kb plus DNA ladder (Invitrogen®), the size of the bands is from bottom to top: 100 bp, 200 bp, 300 bp, 400 bp, 500 bp, 650 bp, 850 bp, 1000 bp, 1650 bp, 2000 bp and increments of 1000 bp up to 12000 bp. [file 1471-2180-14-86-S4.pptx]

## Slide 1
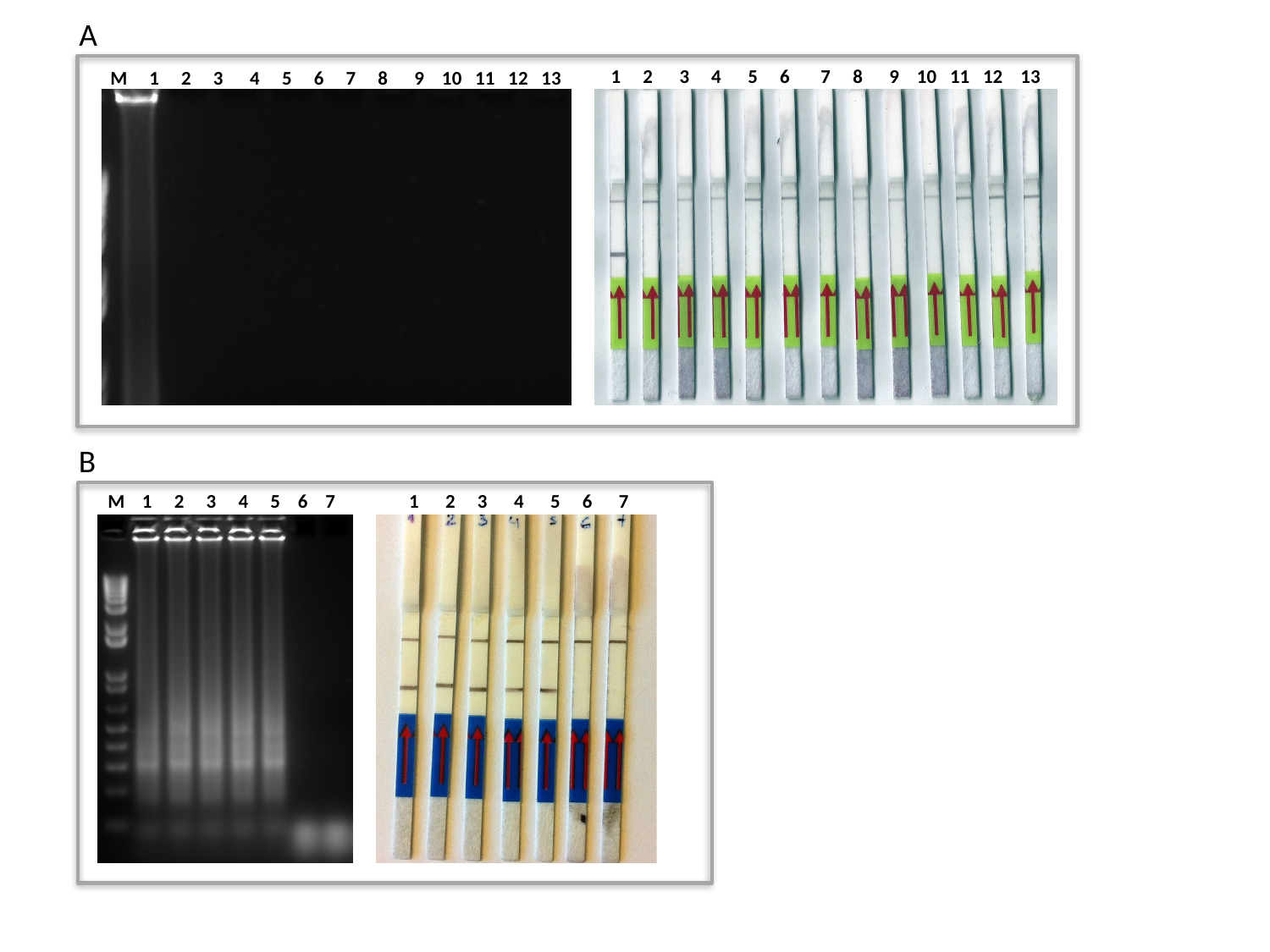

A
1 2 3 4 5 6 7 8 9 10 11 12 13
M 1 2 3 4 5 6 7 8 9 10 11 12 13
B
M 1 2 3 4 5 6 7
 1 2 3 4 5 6 7

Supplement: Additional file 5: Figure S5 — Images of gel electrophoresis and lateral flow dipsticks corresponding to Table 1 and Table 2. A. Images of gel electrophoresis (left) and lateral flow dipsticks (right) corresponding to samples in Table 1. 1. Candidatus Liberibacter asiaticus, 2. Xylella fastidiosa, 3. Xanthomonas campestris pv. campestris, 4. Xanthomonas campestris pv. vesicatoria, 5. Pseudomonas syringae, 6. Botrytis cinerea, 7. Phytophthora citricola, 8. Guignardia citricarpa, 9. Elsinoe fawcettii, 10. Healthy Orange, 11. Healty Citrus limon, 12. Healty Diaphorina citri. B. Images of gel electrophoresis (left) and lateral flow dipsticks (right) corresponding to samples in Table 2. 1. 100 ng DNA, 2. 10 ng DNA, 3. 1 ng DNA, 4. 100 pg DNA, 5. 10 pg DNA, 6. 1 pg DNA, 7. 100 fg DNA. For all gels, M: 1 Kb plus DNA ladder (Invitrogen®), the size of the bands is from bottom to top: 100 bp, 200 bp, 300 bp, 400 bp, 500 bp, 650 bp, 850 bp, 1000 bp, 1650 bp, 2000 bp and increments of 1000 bp up to 12000 bp. [file 1471-2180-14-86-S5.pptx]
